# Development of a novel humanized mouse model to study bronchopulmonary dysplasia

**DOI:** 10.3389/fped.2023.1146014

**Published:** 2023-07-14

**Authors:** Rob Birkett, Janu Newar, Abhineet M. Sharma, Erika Lin, Lillian Blank, Suchitra Swaminathan, Alexander Misharin, Karen K. Mestan

**Affiliations:** ^1^ Department of Pediatrics/Division of Neonatology, Ann & Robert H. Lurie Children’s Hospital of Chicago and Northwestern University Feinberg School of Medicine, Chicago, IL, United States; ^2^ Department of Pediatrics/Division of Neonatology, UC San Diego School of Medicine & Rady Children’s Hospital of San Diego, La Jolla, CA, United States; ^3^ Department of Medicine/Division of Rheumatology, Northwestern University Feinberg School of Medicine, Chicago, IL, United States; ^4^ Department of Medicine/Division of Pulmonary & Critical Care, Northwestern University Feinberg School of Medicine, Chicago, IL, United States

**Keywords:** hematopoietic stem cells, intrauterine inflammation, chorioamnionitis, preeclampsia, preterm birth, neonatal lung disease, fetal monocytes

## Abstract

**Rationale:**

The role of circulating fetal monocytes in bronchopulmonary dysplasia is not known. We utilized a humanized mouse model that supports human progenitor cell engraftment (MISTRG) to test the hypothesis that prenatal monocyte programming alters early lung development and response to hyperoxia.

**Methods:**

Cord blood-derived monocytes from 10 human infants were adoptively transferred into newborn MISTRG mice at p0 (1 × 10^6^ cells/mouse, intrahepatic injection) followed by normoxia versus hyperoxia (85% oxygen × 14 days). Lungs were harvested at p14 for alveolar histology (alveolar count, perimeter and area) and vascular parameters (vWF staining for microvessel density, Fulton's index). Human CD45 staining was conducted to compare presence of hematopoietic cells. Murine lung parameters were compared among placebo and monocyte-injected groups. The individual profiles of the 10 patients were further considered, including gestational age (GA; *n* = 2 term, *n* = 3 moderate/late preterm, and *n* = 5 very preterm infants) and preeclampsia (*n* = 4 patients). To explore the monocyte microenvironment of these patients, 30 cytokines/chemokines were measured in corresponding human plasma by multiplex immunoassay.

**Results:**

Across the majority of patients and corresponding mice, MISTRG alveolarization was simplified and microvessel density was decreased following hyperoxia. Hyperoxia-induced changes were seen in both placebo (PBS) and monocyte-injected mice. Under normoxic conditions, alveolar development was altered modestly by monocytes as compared with placebo (*P* < 0.05). Monocyte injection was associated with increased microvessel density at P14 as compared with placebo (26.7 ± 0.73 vs. 18.8 ± 1.7 vessels per lung field; *P* < 0.001). Pooled analysis of patients revealed that injection of monocytes from births complicated by lower GA and preeclampsia was associated with changes in alveolarization and vascularization under normoxic conditions. These differences were modified by hyperoxia. CD45+ cell count was positively correlated with plasma monocyte chemoattractant protein-1 (*P* < 0.001) and macrophage inflammatory protein-1β (*P* < 0.01). Immunohistochemical staining for human CD206 and mouse F4/80 confirmed absence of macrophages in MISTRG lungs at P14.

**Conclusions:**

Despite the inherent absence of macrophages in early stages of lung development, immunodeficient MISTRG mice revealed changes in alveolar and microvascular development induced by human monocytes. MISTRG mice exposed to neonatal hyperoxia may serve as a novel model to study isolated effects of human monocytes on alveolar and pulmonary vascular development.

## Introduction

Bronchopulmonary dysplasia (BPD) remains the most common chronic lung complication of preterm birth. Although lower gestational age (GA) and birth weight (BW) are independent risk factors, certain endotypes of BPD appear to arise in the fetal stages of lung development. In utero events, compounded by postnatal environmental stressors such as hyperoxia, inflammation and oxidant stress account for the wide variation in pulmonary and overall health outcomes of preterm infants.

During the transition to extrauterine life, stem and progenitor cells in fetal circulation play key roles in early lung injury and repair in response to relative hyperoxia after birth. Fetal monocytes, arising from the fetal liver, give rise to lung macrophages and dendritic cells responsible for inflammatory changes and fibrosis characteristic of BPD (1, 2). The largest subpopulation of monocytes (classical, CD14^+^ CD16^−^) will differentiate and engraft in the lungs as alveolar macrophages (3) but this transition does not occur immediately in humans or mice (4). While it has been reported that monocytes are present in tracheal aspirates of human newborn infants (5, 6), the role of circulating monocytes in lung alveolar and vascular development and their response to hyperoxia during this period is not completely understood. More comprehensive interrogation of monocyte function in an *in vivo* experimental model is needed. Recent advances in humanized animal models have allowed us to better understand the fate and function of human monocytes in complex diseases for which human experimentation is not feasible (6). However, there are no studies reported to date on the effects of chronic neonatal hyperoxia in a humanized mouse model that specifically supports adoptive transfer of monocytes and engraftment of alveolar macrophages. The MISTRG mouse model, in which human GM-CSF and other human cytokines are knocked in to allow robust engraftment of human immune stem and progenitor cells, provides a supportive and novel genetic background upon which to study human BPD.

We recently characterized the gene expression profiles of human fetal monocytes in a large cohort of preterm infants (7). Bulk RNAseq of classical and intermediate monocyte subsets revealed distinct gene expression pathways that appear to be driven by placental inflammatory versus vascular dysfunction. Specifically, fetal monocytes exposed to intrauterine inflammatory processes such as chorioamnionitis upregulate biological pathways related to monocyte activation, chemotaxis and platelet function. Conversely, monocytes exposed to placental vascular disease such as preeclampsia downregulate these processes.

Prior studies of human monocyte transplantation in immunodeficient mice have suggested an overall protective effect of monocytes on long-term alveolarization and pulmonary function (8). Data on earlier alveolarization and vascular development is lacking, specifically at p14 to capture earlier mechanisms relevant to the fetal origins of BPD pathogenesis. This time period is important to understand the transitional influence of circulating monocytes prior to differentiation into alveolar macrophages, which typically occurs after 3 weeks (>p21). As human BPD is a highly heterogenous disease, utilization of a humanized mouse model in which fetal monocytes with distinct human profiles will allow us to identify perinatal influences that cannot be fully recapitulated in traditional animal models. These influences include perinatal processes of placental vascular dysfunction that are associated with known risk factors for BPD such as preeclampsia (9).

In this study, we investigate neonatal lung development in humanized MISTRG newborn mice upon transplantation of human monocytes derived from cord blood of infants representing a wide range of gestational ages and distinct perinatal birth characteristics. The objectives were to evaluate early influences of circulating monocytes on alveolar and pulmonary vascular development, and to test the hypothesis that fetal monocytes from births with specific perinatal profiles drive distinct lung histologic changes in response to hyperoxia.

## Materials and methods

### Patient enrollment

**Figure 1:**
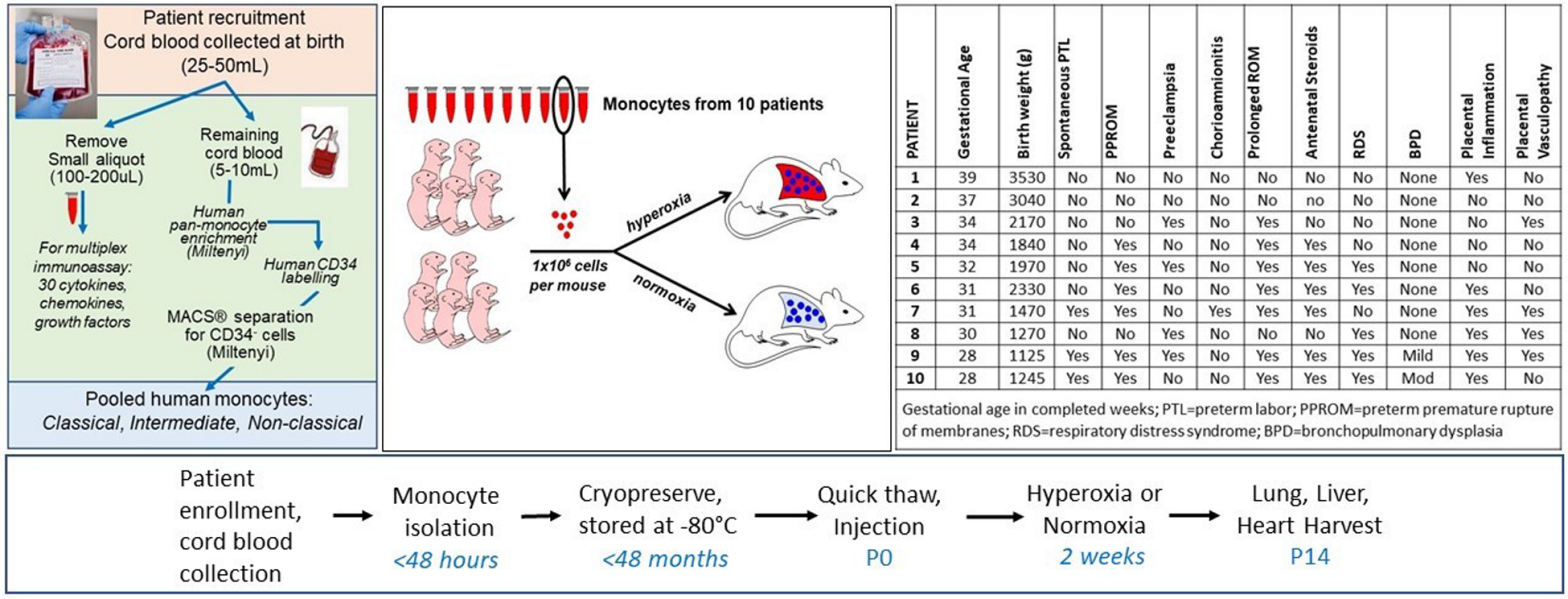
Overview and timeline of experiments. Cord blood monocytes isolated through the workflows and protocols depicted on the far left from 10 individual patients were injected into P0 mice via intrahepatic injection. Mice were immediately placed in hyperoxia (85%) or normoxia (21%) for 14 days. Characteristics of the 10 patients are shown in the table (far right). For 7 of the 10 patients, at least 6–8 pups were injected with patient-specific monocytes. For patients #4, 5, and 7, only 2 pups injected with these monocytes were available for the normoxia group due to smaller paired litters available and lower than expected monocyte counts at the time of sample thaw. In addition, for patients #8 and 9, at least 3 pups were injected for normoxia and hyperoxia groups but all pups died in hyperoxia and 3 total died in hyperoxia before P14, leaving only 2 pups each in the hyperoxia group for lung parameter analyses at P14.

Ten mothers and their newborn infants were included in this study (Figure 1). Participants were prospectively enrolled through an ongoing biorepository in which all women delivering a liveborn infant at Prentice Women's Hospital (Chicago, IL) are eligible. Informed consent was obtained from all participants prior to participation. The study was approved by the Institutional Review Board of Northwestern University. The 10 births were selected based upon the availability and quality of monocytes recovered from cord blood at birth, including quantity of viable monocytes sufficient to conduct each experiment injecting 1 × 10^6^ monocytes per mouse pup using a single patient per litter (e.g., for 1 litter of 6–8 pups each, a patient with at least 6–8 million monocytes available for injection into 6–8 pups was chosen, so that half of each litter could be placed in normoxia and the other half in hyperoxia) (Figure 1). In cases of smaller litters and quantity of monocytes available at the time of injection, more than or fewer than 3–4 pups per exposure group were studied. Infants with known congenital anomalies, infections and genetic syndromes were excluded.

### Clinical data

Maternal and infant baseline and clinical data were obtained using standardized protocols of the larger birth cohort, with definitions as previously published by this group (7, 10). GA at birth was recorded as completed weeks and further categorized according to CDC classifications: full term birth was defined as >37 completed weeks, moderate or late preterm birth was defined as 32–36 completed weeks, and very preterm was defined as <32 completed weeks (11, 12). Preeclampsia was defined according to American College of Obstetricians and Gynecologists' (ACOG) criteria, and included other hypertensive disorders of pregnancy, such as eclampsia and hemolysis, elevated liver enzymes, low platelets (HELLP) syndrome (13).

### Cord blood collection

Delivery staff collected venous cord blood at time of birth into a cord blood bag or EDTA tube as previously described (10, 14). Cord blood specimens were stored at 4°C and monocyte isolation was performed within 48 h of delivery. Corresponding cord blood plasma from all 10 patients was separated by refrigerated tabletop centrifuge and pipetted into aliquots of 100–200 ul each, and stored at −80°C until multiplex immunoassay (see below).

### Monocyte isolation, enrichment, cryopreservation and thaw

Cord blood specimens were spun down at 1,400 rpm for 10 min (Figure 1). Plasma and anticoagulant were removed. Red blood cells were lysed for 15 min at room temperature in the dark, using BD Pharm Lyse (BD Biosciences, San Jose, CA) at 10 ml lysis buffer per 1 ml cord blood. PBS was added to the tube(s) and centrifuged at 1,400 rpm for 5 min. After removing the lysis buffer solution, the cells were washed with 2% BSA in PBS to remove residual lysis buffer. Monocytes were enriched using a human pan-monocyte enrichment kit according to manufacturer's protocol (Pan Monocyte Isolation Kit, Catalog#: 130-096-537, Miltenyi Biotec, Germany). Non-monocytes were magnetically labeled with a cocktail of biotin-conjugated antibodies. The cell solution was filtered through an LS column (for up to 10^8^ magnetically labeled cells) attached to a magnetic field of a MACS® Separator. This ensured a quick and gentle separation of magnetically labeled cells from monocytes. Following monocyte enrichment, the enriched monocyte solution was treated with CD34 microbeads to magnetically label CD34+ cells with a human CD34 Microbead Kit (Catalog#: 130-046-702, Miltenyi Biotec, Germany). The treated monocyte solution was filtered through an MS column (for up to 10^7^ magnetically labeled cells) attached to a magnetic field of a MACS® Separator. The CD34+ cells adhered to the MS column, while the monocytes were eluted. The cells were counted to obtain cell concentration and viability (Bio-Rad, Hercules, CA).

The monocytes were frozen in 2 ml tubes using Cell Therapy Systems (CTS) Synth-a-Freeze medium (Gibco, Thermo Fisher, Waltham, MA) in Thermo Scientific™ Mr. Frosty™ freezing containers (Thermofisher, Waltham, MA). The system is designed to achieve a rate of cooling close to −1°C/min to allow an optimal rate for cell cryopreservation. Cryopreserved cells were placed at −80°C and stored until use. All cells were used within 24 months of cryopreservation (median storage time = 12 months). Given the uncertainty and variability of when the archived cells would be needed for the multiple experiments, we opted to keep all monocyte aliquots stored together in a single −80°C freezer until thaw to avoid variations in freeze methods among the patient samples. This approach was supported by past literature showing no significant difference in cell viability and function of peripheral blood stem cells stored up to 5 years using either conventional liquid nitrogen or mechanical freezer (15). A standardized “quick thaw” protocol was adopted from our own experience with monocytes and preliminary studies supporting the highest viability of monocytes upon thaw. Briefly, cryopreserved monocytes were removed from cryovials and placed in a 37°C water bath for 30 s. Media (10% FBS in RPMI + pen strep) warmed at 37°C was slowly added and the cell suspension was transferred to a 15 ml conical tube to a volume of 10 ml media. Cell viability and count was again determined upon thaw. Only patient samples with at least 80% viability were used for the adoptive transfer experiments (mean cell viability = 88%).

### Mouse experimental conditions

The Institutional Animal Care and Use Committee at Northwestern University approved all animal procedures. MISTRG mice were procured from Jackson Lab (JAX 017712), and maintained on 0.27 mg/ml Baytril as previously described (6). Timed matings were conducted to ensure at least 2 litters of at least 6 pups each litter, and equal numbers of male and female pups, for each experiment using at least 2 patient samples and placebo (PBS) at a time. At p0, litters were examined for the presence of milk spots and to ensure dams were caring for the pups. Cryopreserved monocytes were thawed (see above) and resuspended at a concentration of 1 × 10^6^ monocytes per 30 ul of PBS and administered by intrahepatic injection on p0. An equal volume of PBS was used for placebo injections (PBS group, *N* = 17 mice). Mice were kept at room air (21% O_2_) or at hyperoxia (85% O_2_) in a Plexiglas chamber (Biospherix, Lacona, NY) for 14 days. Nursing dams were rotated every 24 h to prevent oxygen toxicity to adult animals.

### Harvested tissues

P14 pups were euthanized according to the procedures outlined by the panel on euthanasia of the American Veterinary Medical Association. The lungs, hearts, and livers were harvested. The lungs were inflated to 25 cm H_2_O with 10% formalin. Hearts were harvested at time of euthanasia and Fulton's index determined as previously described (16). Tissue processing and sectioning was performed by the Histology Core at Stanley Manne Children's Research Institute.

### Lung morphometry

Lungs were sectioned and stained with hematoxylin and eosin. Images were taken with an Olympus BX40 microscope. Six nonoverlapping images were taken per animal and alveolar area and counts were measured with ImagePro (Media Cybernetics, Rockville Maryland). Mean/median values were reported for each animal. All slides were coded and randomly analyzed by a single examiner (RB) masked to the original experimental conditions, patient group assignments and treatments/exposures.

### Small vessel density

Sections were incubated with von Willebrand Factor (vWF) primary antibody (Dako, Carpenteria, CA). 6–8 images/animal were randomly captured under 10× magnification. Small vessels (<100 µm) were counted and averaged per animal in masked fashion as described above for lung morphometry.

### Immunohistochemistry

Anti-human CD45 (Cell Signaling, Danvers, MA) at a dilution of 1:250 and 3, 3-diaminobenzidine (DAB; Vector Labs, CA) was used to stain lung and liver tissues. CD45+ expression was observed and recorded at 10×. Immunohistochemistry staining for anti-human CD206 (Sigma-Aldrich, MO) was performed by the Northwestern Pathology Core Facility to identify presence of human alveolar macrophages in representative lung samples. Staining for mouse-specific macrophages was performed using anti-mouse F4/80 (BD Biosciences) with biotin labelled secondary antibody. Lung tissue from a non-humanized mice strain (C57BL/6J, wildtype adult mice 12 months, provided by Eniko Sajti) was used as a positive control.

### Identification of human alveolar macrophages at p56

Single cell suspension of mouse lung harvested at p56 was prepared according to an established protocol (17). Briefly, the mouse lungs were perfused with PBS through the right ventricle. After mincing the lung tissue with scissors, the tissue was transferred to C-tubes (Miltenyi, Auburn, CA). The tissue was digested in HBSS with 1 mg/ml Collagenase D and 0.1 mg/ml DNase I (Roche, Indianapolis, IN) and dissociated with a GentleMACS dissociator (Miltenyi). The cell suspension was passed through a 40 um filter before being MACS enriched for human CD45 (Miltenyi) according to manufacturer's instructions. The cells were stained with HLA-DR, mCD45, hCD206, hCD14 (BD Biosciences, Franklin Lakes, NJ), and SYTOX Green (Thermo Fisher, Waltham, MA). FACS was performed on a BD FACSAria SORP Cell Sorter at the Northwestern Robert H. Lurie Cancer Center Flow Cytometry Core Facility. HLA-DR+, hCD206+, hCD14+, mCD45- viable cells were sorted and subsequently stained for H&E following cytospin to generate the images shown in Figure 11.

### Multiplex immunoassays

Simultaneous measurement of 30 analytes was performed by sandwich immunoassays using Luminex xMAP platform in magnetic bead format: EGF, Eotaxin, FGF-2, FLT-3l, Fractalkin, granulocyte colony stimulating factor (G-CSF), GM-CSF, IFN-γ, IL-1α, IL-1β, IL-1RA, IL-2, IL-3, IL-4, IL-6, IL-8, IL-10, IL-12p40, IL-12p70, IL-13, IL-17A, IP-10, monocyte chemoattractant protein-1 (MCP-1), MCP-3, macrophage inflammatory protein (MIP)-1α, MIP-1β, TGFα, TNFα, TNFβ, VEGFA. The multiplexed assay beads were obtained from a commercially available kit (EMD Millipore, MA). Plasma samples were thawed on ice and prepared in 1:1 dilution and analyzed according to manufacturer's instructions. All samples were run in duplicate with standard curves for each marker and controls on each plate. Only the analytes that fell within the limits of detection for at least 90% of the patient samples were included in linear regression analyses.

### RT-qPCR

RNA Isolation: Total cellular RNA was isolated using the Quick-RNA Mini-prep kit (Direct-zol, R2052, ZYMO Research, Irvine, CA, USA) according to the manufacturer's instructions. The concentration of total RNA was determined by Qubit® 2.0 Fluorometer. cDNA synthesis: Complementary DNA (cDNA) was synthesized using Sensiscript RT Kit (QIAGEN) and random hexamers (QIAGEN) according to the manufacturer's recommendations. Briefly, 40 ng of total RNA was added to the master mix containing 2 µl of 10× buffer RT, 2 µl of dNTP mix (5 mM each of dNTP), 10 µm of random hexamers, and 1 µl RNase inhibitor (10 units/µl), 1 µl Sensiscript Reverse Transcriptase and volume was made up by RNase-free water in a PCR tubes. The PCR tube were incubated at 37°C for 60 min for cDNA synthesis. Quantitative RT-PCR TaqMan Assays: Real-time PCR was performed using a CFX-96 (Bio-Rad, Hercules, CA) System and TaqMan Fast Advanced Master Mix (Applied Biosystems). TaqMan® Gene Expression Assay IDs used for this assay are Human CCL2 (Hs00234140_m1), Human CCL4, (Hs99999148_m1) Human VEGFA (Hs00900055_m1), Human GAPDH (Hs02758991_g1), Mouse CCL2 (Mm00441242_m1), Mouse CCL4 (Mm00443111_m1), Mouse VEGFA (Mm00437306_m1) Mouse GAPDH (Mm99999915_g1). Real-time PCR amplification was performed using a total volume of 10 µl that contained 1 µl cDNA (40 ng), 5 µl TaqMan Fast Advanced Master Mix, 0.5 µl of each TaqMan Gene Expression Assay, and 3.5 µl ultrapure DNase-free water. The cycle parameters were as follows: UNG incubation at 50°C for 2 min, polymerase activation at 95°C for 20 s, denaturation at 95°C for 3 s and then annealing and extension at 60°C for 30 s. Ct values were calculated, defined as the number of cycles required for the fluorescent signal of a sample to cross the threshold line, and was inversely proportional to the amount of target nucleic acid in the sample (18).

### Statistical analysis

Patient demographics and clinical characteristics were compared using ANOVA or Kruskal Wallis for continuous variables and *X*^2^ or Fisher exact tests for categorical data. Lung tissue parameters from mice were reported as mean or median depending upon normality of distribution, and groups were compared using parametric or non-parametric tests where appropriate, with *post hoc* tests and adjustment for multiple comparisons with Bonferroni correction. Multivariate linear regression models were used to measure the correlations (beta-coefficients) between cord blood plasma cytokine/chemokine levels and lung parameters, with log-transformation of all continuous variables, adjustment for pertinent clinical variables, and stratification of the models by hyperoxia exposure (yes/no). *P* < 0.05 was considered statistically significant. Statistical analyses were performed using STATA/IC version 13.0 (StataCorp, College Station, TX). Graphs were prepared using GraphPad Prism 8.0 (San Diego, CA).

## Results

### The patient sample represented a wide range of gestational ages and clinical features

Relevant baseline demographics and clinical characteristics of the ten mothers and their infants are shown in Table 1. GA at birth ranged from 28 to 39 completed weeks (Mean GA: 32.9 ± 3.6 weeks). BW ranged from 1,125 to 3,530 grams (Mean BW: 1,999 ± 798 grams). Among the 10 patients, 2 were full term and 8 were born preterm: 3 were moderate to late preterm (32–34 completed weeks) and 5 were very preterm (28–31 completed weeks).

**Table 1:** Perinatal characteristics of the patient sample.

	All births *N* = 10	Full term *N* = 2	Late preterm *N* = 3	Very preterm *N* = 5
Gestational age, weeks				
(mean ± SD)	32.9 ± 3.6	38.4 ± 1.9	33.7 ± 1.2	30.3 ± 1.8
Birth weight, grams	1,999 ± 798	3,285 ± 346	1,993 ± 166	1,488 ± 487
Birth weight-for-GA, percentile	49.3 ± 27.4	59.4 ± 10.6	37.9 ± 21.4	52.0 ± 35.7
Maternal age, years	30.5 ± 5.9	32.0 ± 1.4	26.3 ± 7.6	32.4 ± 5.4
Infant sex, *n* (%)				
Male	6 (60)	1 (50)	2 (67)	3 (60)
Female	4 (40)	1 (50)	1 (33)	2 (40)
Rupture of membranes (ROM)				
Spontaneous	6 (60)	1 (50)	2 (67)	3 (60)
Artificial	4 (40)	1 (50)	1 (33)	2 (40)
Mode of delivery				
Vaginal	8 (80)	2 (100)	3 (100)	3 (60)
Cesarean section	2 (20)	0 (0)	0 (0)	2 (40)
Antenatal steroids (complete)				
No	4 (40)	2 (100)	1 (33)	1 (20)
Yes	6 (60)	0 (0)	2 (67)	4 (80)
Spontaneous preterm labor				
No	7 (70)	2 (100)	3 (100)	2 (40)
Yes	3 (30)	0 (0)	0 (0)	3 (60)
Preterm premature ROM				
No	4 (40)	2 (100)	1 (33)	1 (20)
Yes	6 (60)	0 (0)	2 (67)	4 (80)
Prolonged ROM				
No	3 (30)	2 (100)	0 (0)	1 (20)
Yes	7 (70)	0 (0)	3 (100)	4 (80)
Chorioamnionitis				
No	9 (90)	2 (100)	3 (100)	4 (80)
Yes	1 (10)	0 (0)	0 (0)	1 (20)
Preeclampsia				
No	6 (60)	2 (100)	1 (33)	3 (60)
Yes	4 (40)	0 (0)	2 (67)	2 (40)

### Hyperoxia exposure resulted in increased alveolar simplification and decreased pulmonary microvascular development in the MISTRG mice at P14, independent of monocyte versus PBS injection at P0

**Figure 2:**
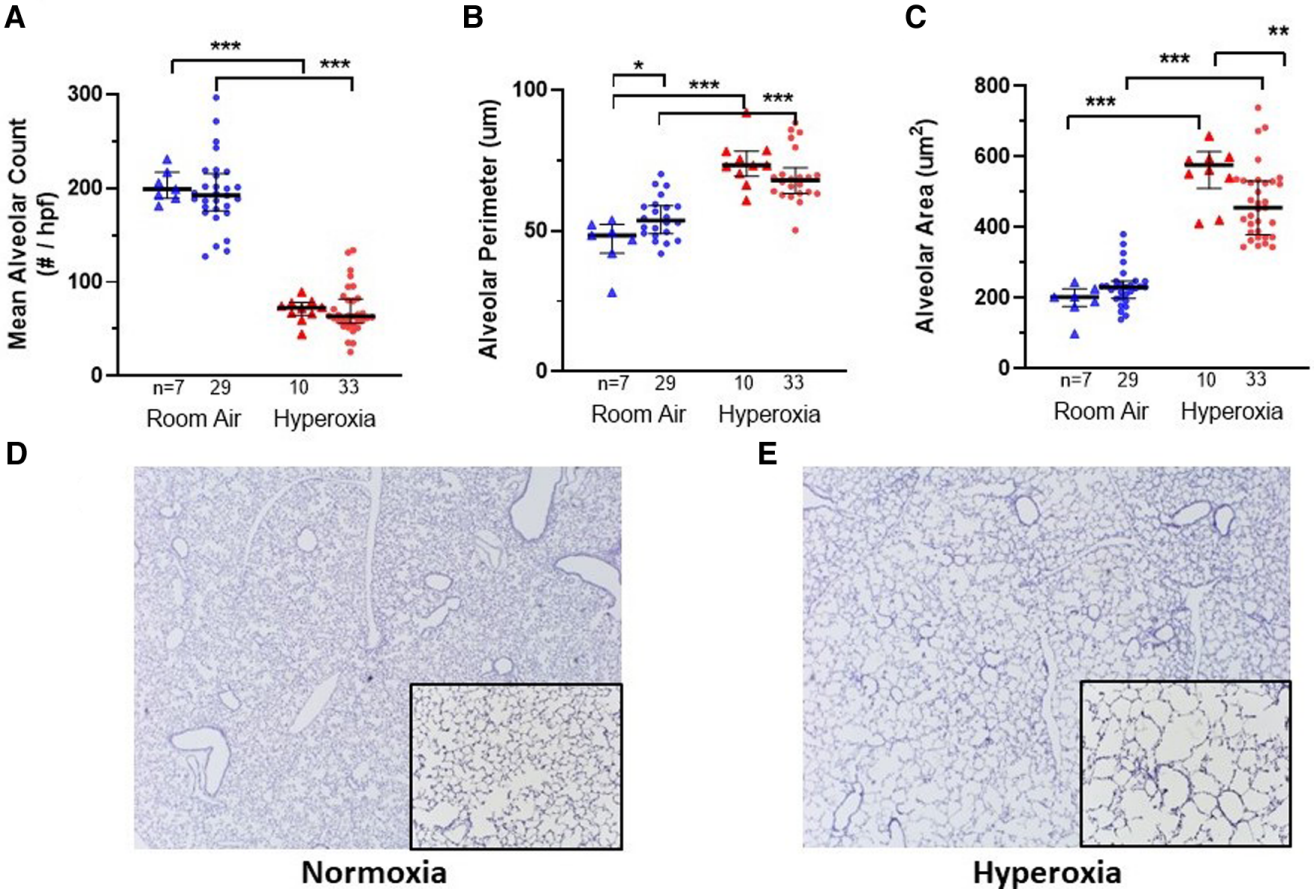
Lung alveolar morphometry and representative images at P14 according to monocyte versus placebo treatment on p0 and normoxia versus hyperoxia exposure (p0–p14). (**A–C**) Summary of alveolar count, perimeter and area, respectively. Individual datapoints represent each mouse: triangles represent PBS-injected and dots represent monocyte-injected pups. Blue indicates normoxia and red indicates hyperoxia exposure. At p14, mean alveolar area (**C**) was decreased in mice treated with human monocytes and exposed to hyperoxia. Mean alveolar perimeter (**B**) but not alveolar count (**A**) was increased with monocyte transplantation followed by room air. *P < 0.05, **P < 0.01 and ***P < 0.001. (**D–E**) Representative images of mouse lungs after 14 days of normoxia (**D**) versus hyperoxia showing typical alveolar simplification (**E**). Images stained for human CD45 with counterstaining. Images taken at 10× with 20× inlay.

**Figure 3:**
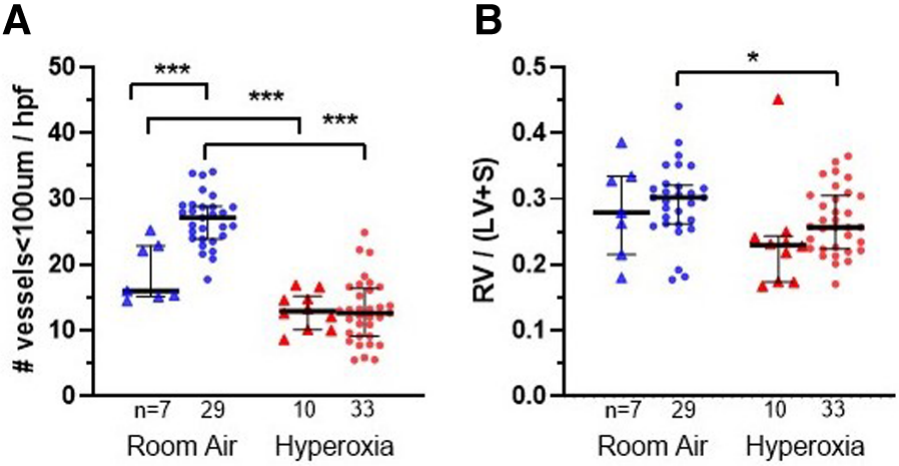
Lung vascular parameters and representative images at P14 according to monocyte versus placebo treatments and normoxia versus hyperoxia exposures. Individual datapoints represent each mouse: triangles represent PBS-injected and dots represent monocyte-injected pups. Blue indicates normoxia and red indicates hyperoxia exposure. (Panel **A**) small vessel density (measured by vonWillebrand factor stain) was increased in room air-exposed mice treated with monocytes. (Panel **B**) Fulton's index (ratio of right ventricular weight/left ventricle + septum) was decreased in monocyte-injected group with hyperoxia as compared with normoxia. *P < 0.05, **P < 0.01 and ***P < 0.001.

**Figure 4:**
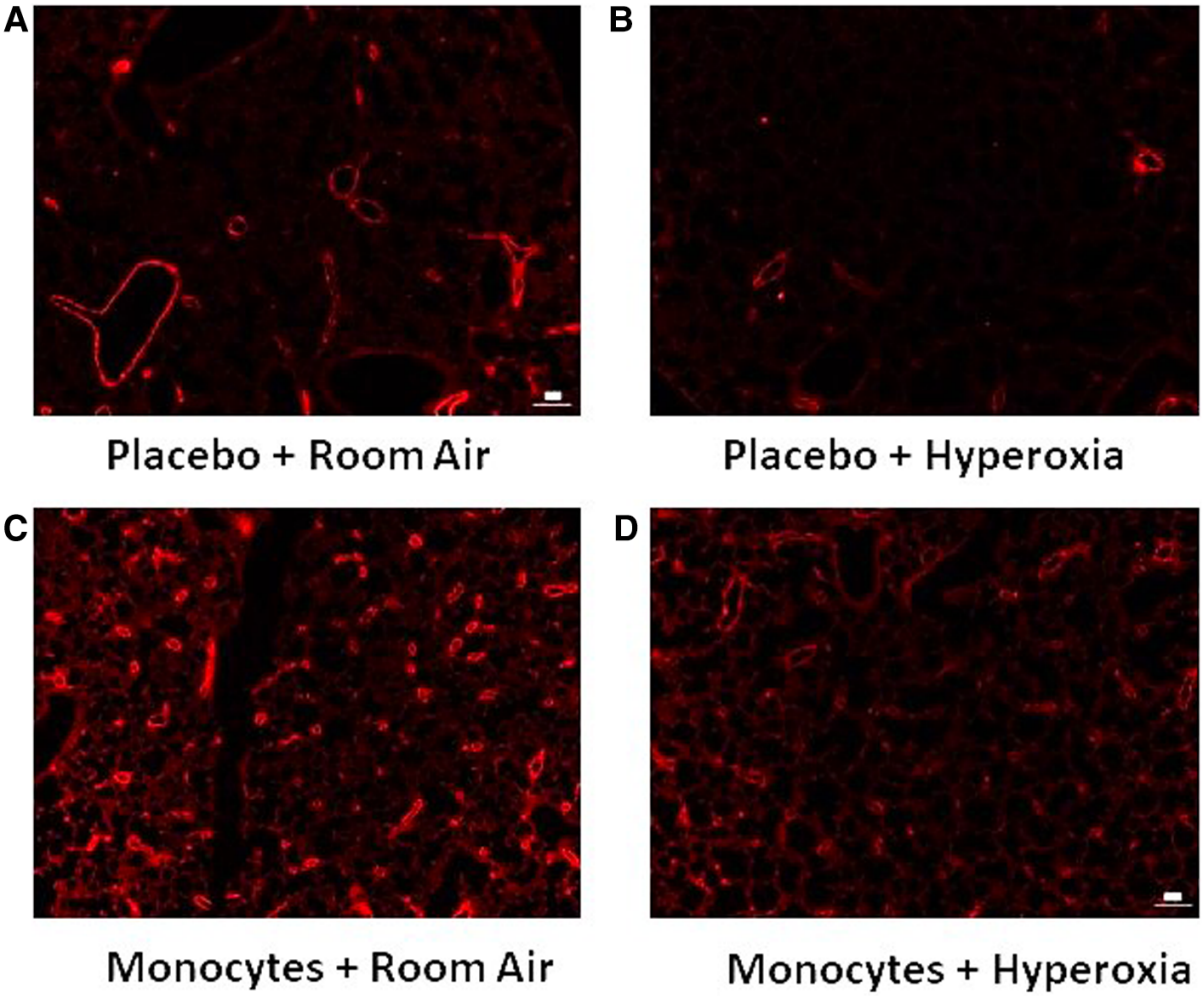
Representative immunofluorescent images with vWF identifying microvessel density. Note the relative paucity of microvessels (vessel diameter <100 um) in mice treated with placebo (**A–B**) versus intrahepatic injection of monocytes (**C–D**).

Figures 2, 3 show the individual datapoints for each of the 79 mice harvested at P14, after injection at P0 with either PBS (*n* = 17) or human monocytes from 10 patient donors (*n* = 62) followed by either 14 days of hyperoxia (*n* = 43) vs. room air (*n* = 36). Across most all histologic parameters and independent of PBS or monocyte injection, hyperoxia resulted in decreased mean alveolar count (*P* < 0.001 for both PBS and monocyte-injected groups), increased alveolar perimeter (*P* < 0.001), and increased alveolar area (*P* < 0.001) (Figure 2). Minor changes in alveolar patterns included a decrease in alveolar area among the monocyte-injected group versus placebo (*P* = 0.007; Figure 2C) suggesting that the monocytes attenuated alveolar simplification due to hyperoxia. Hyperoxia also induced the expected changes of decreased microvessel counts in the lungs, as indicated by vWF staining, (see Figure 4 for representative images) in both PBS and monocyte-injected mice (Figure 3A; *P* < 0.001).

### Injection of mice with human monocytes at P0 resulted in increased microvessel count at P14

Figure 3A shows the median microvessel counts per high power field (hpf), as identified by vWF staining. Median vessel count was significantly increased in lungs of pups treated with monocytes as compared with PBS (26.7 ± 0.73 vs. 18.8 ± 1.7 vessels per lung field; Figure 3A). As mentioned above, hyperoxia exposure attenuated the differences in microvessel count between PBS and monocyte-treated mice. However, among the monocyte-treated group, there was a significant decrease in microvessel density with hyperoxia to levels similar to the PBS-treated hyperoxia group (26.7 ± 0.73–12.9 ± 0.83 vessels per lung field; normoxia to hyperoxia, respectively; *P* < 0.001). Collectively, these findings suggest that monocytes play a role in early pulmonary microvascular development, which is attenuated by hyperoxia.

### Hyperoxia exposure resulted in decreased pulmonary hypertension at P14

While RV/LV + S ratios (Fulton's index) were expected to increase with hyperoxia as a measure of pulmonary hypertension in other mouse models (19, 20), the trend appeared overall reversed in the MISTRG mice (Figure 4B). With the exception of two outlying datapoints (Fulton index = 0.45 in one hyperoxia-exposed PBS-treated mouse and 0.44 in one room air-exposed monocyte-treated mouse) the majority of values were <0.4 overall. Regardless, either with or without these outliers included, there was a significant overall decrease in mean Fulton's index with hyperoxia when PBS and monocyte-treated mice were analyzed together (mean index = 0.29 ± 0.01 vs. 0.26 ± 0.01; normoxia versus hyperoxia, respectively; *P* = 0.01). Stratification by PBS versus monocyte-treated groups revealed that Fulton's index was significantly decreased with hyperoxia in the monocyte-treated group (*N* = 62; mean index = 0.30 ± 0.01 vs. 0.27 ± 0.01; normoxia versus hyperoxia; *P* = 0.03) but not in the PBS-treated group (*N* = 17; 0.28 ± 0.03 vs. 0.24 ± 0.03; *P* = 0.24).

### Among the mice injected with human monocytes, there was variability in lung and vascular parameters at baseline and with hyperoxia that could be accounted for by distinct patient-specific features of the monocytes

**Figure 5:**
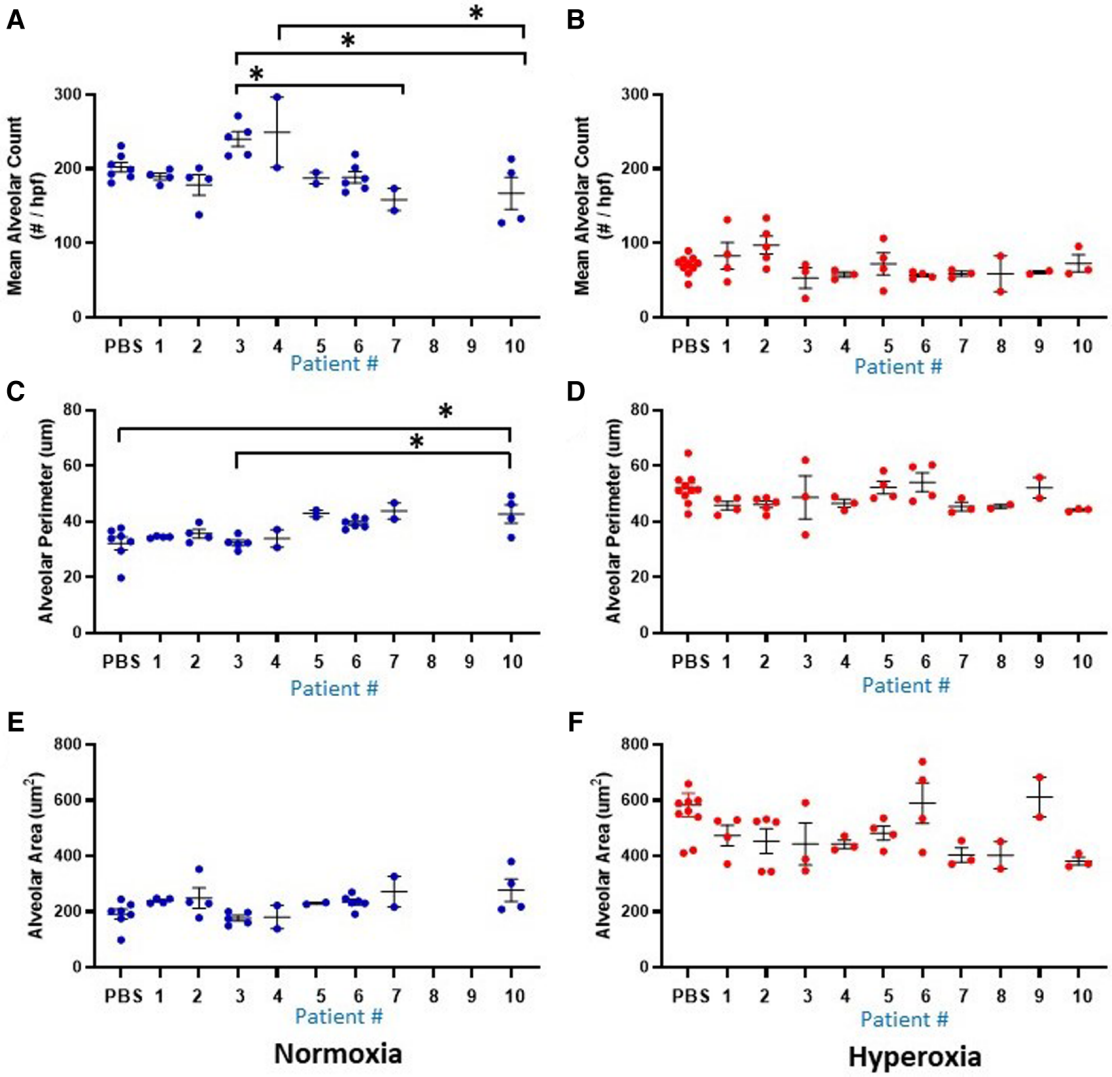
Lung alveolar morphometry data at P14 according to the 10 individual patients (1-10) and placebo (PBS). Individual datapoints represent mean/median values from 6 non-overlapping fields from each mouse for: alveolar count (**A–B**), alveolar perimeter (**C–D**) and alveolar area (**E–F**). Blue dots indicate normoxia and red dots indicate hyperoxia exposure. Data were analyzed using ANOVA with Bonferroni correction and post hoc analysis to identify patient-specific differences among groups. *P < 0.05.

**Figure 6:**
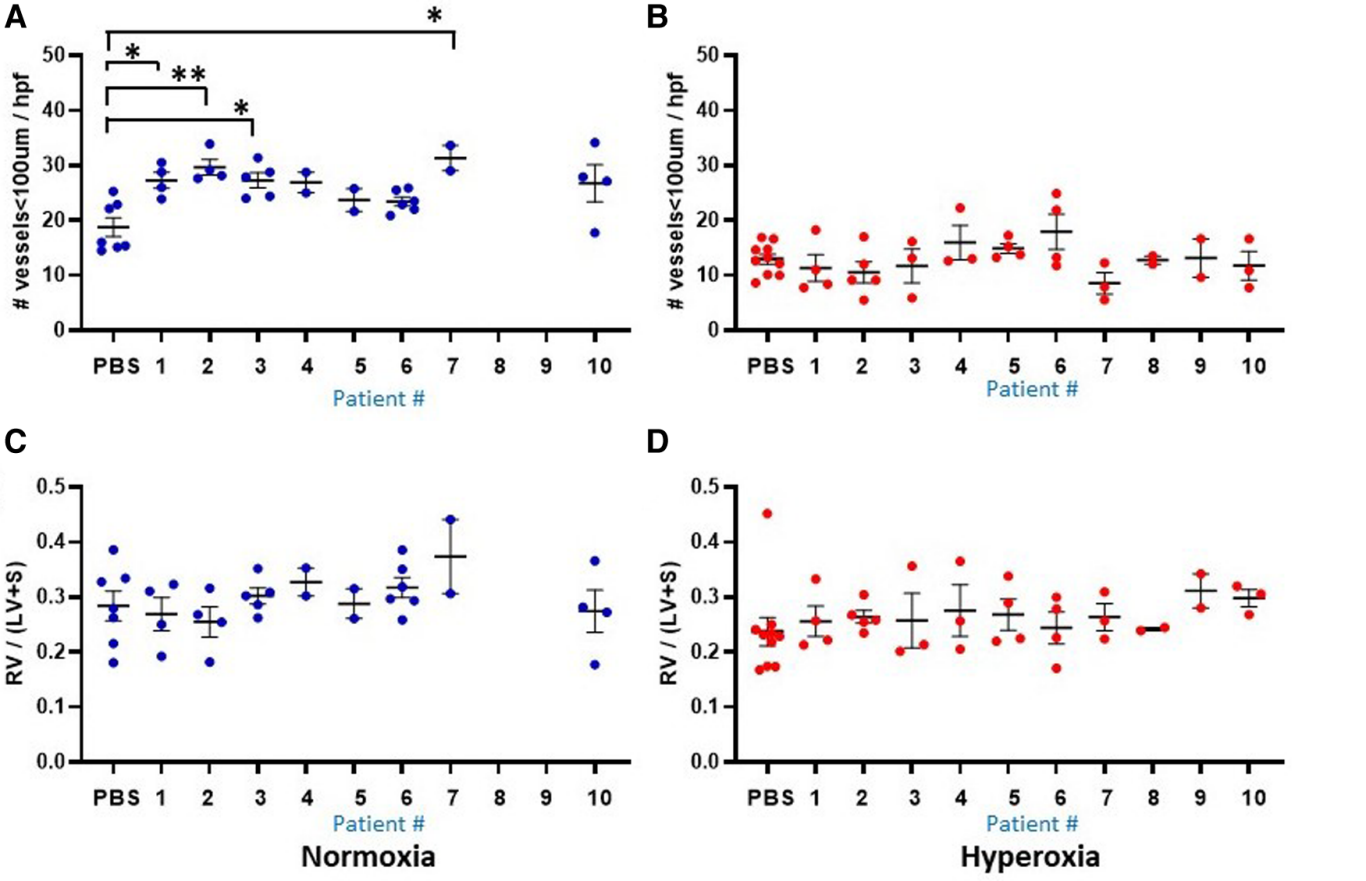
Lung vascular parameters at P14 according to the 10 individual patients (1–10) and placebo (PBS). Individual datapoints represent each mouse for: mean microvessel count (<100um) from 6-8 images (**A–B**), and Fulton's index (**C–D**). Blue dots indicate normoxia and red dots indicate hyperoxia exposure. Data were analyzed using ANOVA with Bonferroni correction and post hoc analysis to identify patient-specific differences among groups. *P < 0.05; **P < 0.01.

Figure 1 delineates the clinical profiles data from all 10 infants of the patient sample, numbered as patient 1 through 10. Figures 5, 6 show the humanized mouse alveolar data according to each individual patient, numbered 1 through 10 corresponding to Figure 1. The individual datapoints representing each mouse demonstrate that there was variability among lung and vascular parameters, suggesting that individual patient characteristics of the 10 infants played a role in these early lung developmental findings. For example, as shown in Figure 5A mean alveolar count was significantly decreased in mice injected with monocytes from very preterm infants (patients 7 and 10) as compared with moderate and late preterm infants (patients 3 and 4). Similarly, alveolar perimeter was increased in mice injected with monocytes from very preterm infant (patient 10) as compared with PBS and a late preterm infant (34 weeks) exposed to preeclampsia (patient #3). Overall, there were significant differences in alveolar count (*P* = 0.001 in normoxia group; *P* = 0.007 in hyperoxia group), perimeter (*P* = 0.002 in normoxia group) and area (*P* = 0.01 in normoxia group) when comparing parameters according to the origin of the monocytes from full term, moderately preterm and very preterm infants by ANOVA with *post hoc* testing. Table 2 summarizes the changes in lung parameters according to the 3 gestational age groups, stratified by normoxia and hyperoxia conditions.

**Table 2:** MISTRG lung parameters according to type of patient monocyte injected.

	Alveolar count	Alveolar perimeter	Alveolar area	Lung microvessel count	Lung CD45+ cell count
Normoxia exposure					
Gestational age groups					
Full term (*n* = 8 mice)	184.1 ± 20.0	35.0 ± 2.2	244.4 ± 48.9	28.5 ± 3.0	9.4 ± 5.2
Moderate preterm (*n* = 9 mice)	230.5 ± 37.8	35.1 ± 5.1	189.6 ± 35.0	26.4 ± 3.0	19.4 ± 14.1
Very preterm (*n* = 12 mice)	176.5 ± 29.7*	41.2 ± 4.3**	255.4 ± 55.3*	25.8 ± 4.9	10.3 ± 9.9
Preeclampsia					
No (*n* = 22 mice)	185.8 ± 7.5	38.3 ± 1.0	224.6 ± 11.7	26.9 ± 0.90	9.2 ± 1.7
Yes (*n* = 7 mice)	225.2 ± 12.0*	35.4 ± 2.1	192.1 ± 12.0*	26.25 ± 1.2	25.0 ± 4.6***
Hyperoxia exposure					
Gestational age groups					
Full term (*n* = 9 mice)	91.0 ± 30.0	46.0 ± 2.6	462.8 ± 84.4	10.9 ± 4.2	6.7 ± 4.6
Moderate preterm (*n* = 10 mice)	61.9 ± 22.5*	49.5 ± 7.4	458.7 ± 72.2	14.3 ± 4.1	8.5 ± 4.4
Very preterm (*n* = 14 mice)	61.5 ± 14.1*	48.6 ± 5.8	482.0 ± 131.5	13.2 ± 5.4	3.8 ± 4.3*
Preeclampsia					
No (*n* = 22 mice)	73.3 ± 5.4	47.2 ± 1.0	464.2 ± 22.0	12.7 ± 1.2	5.7 ± 1.0
Yes (*n* = 11 mice)	62.3 ± 7.1	50.0 ± 2.2	480 8 ± 31.2	13.3 ± 1.0	6.7 ± 1.6

* *P* < 0.05.

** *P* < 0.01.

*** *P* < 0.001 vs. full term or no preeclampsia using ANOVA or student's *t*-test.

**Figure 7:**
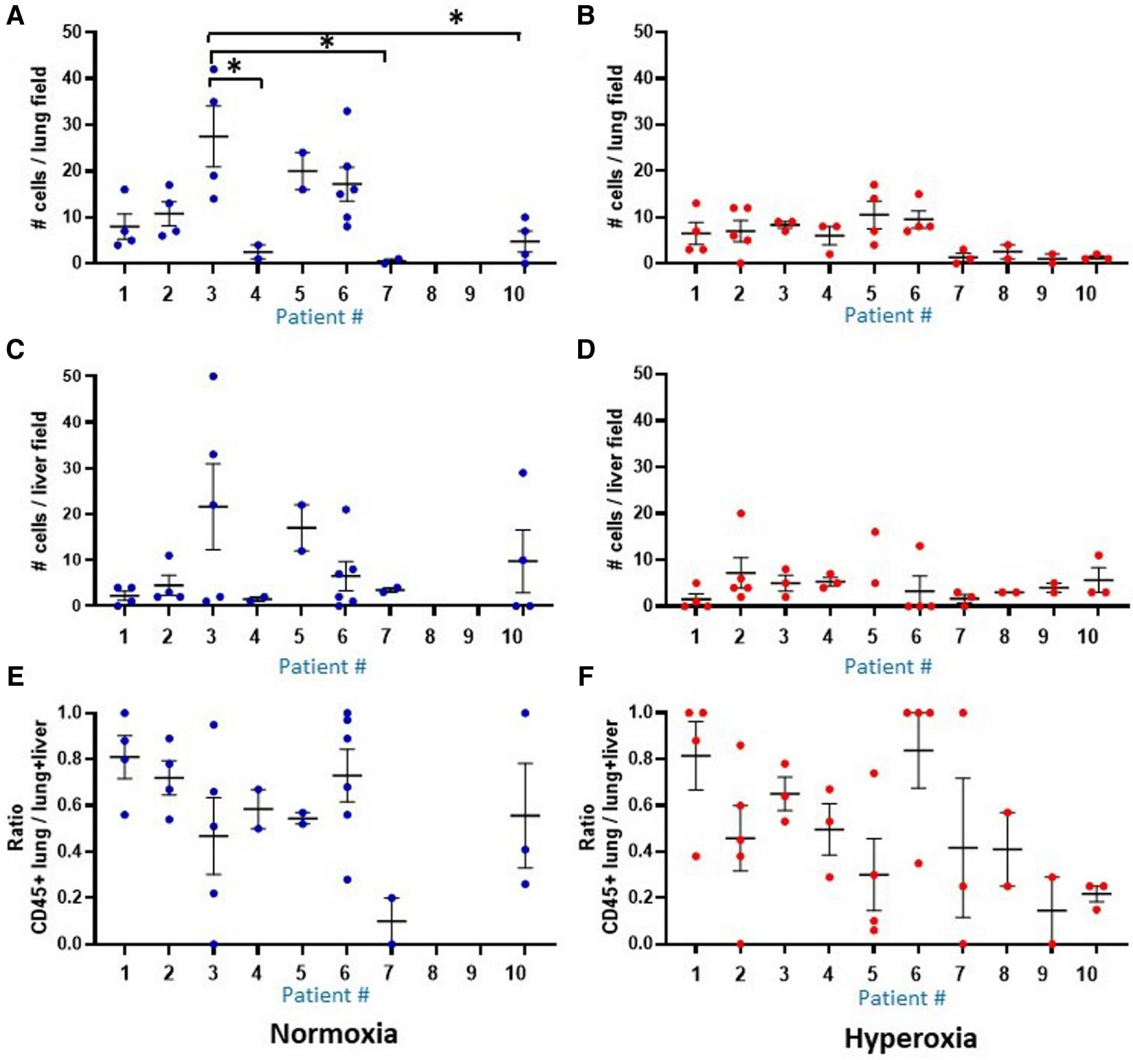
Comparison of human CD45 cell counts in MISTRG lung and liver at P14. Individual datapoints represent each mouse: blue indicates normoxia and red indicates hyperoxia exposure. Anti-human CD45 antibody staining was used to quantify human CD45+ hematopoietic cells circulating in the lungs at p14. (Panel **A,B**) Overall, median human cell count was decreased with hyperoxia exposure in mice treated with the human monocytes. Analysis of liver cell counts (Panel **C,D**) and ratio of lung to lung + liver counts per each mouse (**E,F**) revealed no differences in presence of human cells within the 10 patient groups, and no differences between hyperoxia versus normoxia. Missing data in normoxia (blue) for patients 8 and 9 indicate that all pups died prior to p14 and could not be harvested. *P < 0.05.

Another prominent covariate of preterm birth in this patient sample was preeclampsia, in which 4 of the 10 infants were born to mothers with preeclampsia (patients 3, 5, 8 and 9). Mean alveolar count was increased and alveolar area was decreased in room air-exposed mice injected with monocytes transplanted from preeclamptic births as compared with non-preeclamptic births (Table 2). Comparison of parameters and all under hyperoxic conditions yielded no significant differences. Of note, the mice injected with monocytes from the very preterm infants exposed to preeclampsia (Patients 8 and 9) all died before p14 under normoxic conditions (Figures 5A,C,E), while the mice transplanted with these monocytes under hyperoxia survived to p14 (Figures 5B,D,F). The lung alveolar parameters (Figure 5), vascular findings (Figure 6) and lung CD45 counts (Figure 7) in these mice tended to be similar to other hyperoxia-exposed mice injected with monocytes from very preterm infants (patients 6, 7, and 10).

### Human CD45+ hematopoietic cells are present in MISTRG lungs and liver at P14

**Figure 8:**
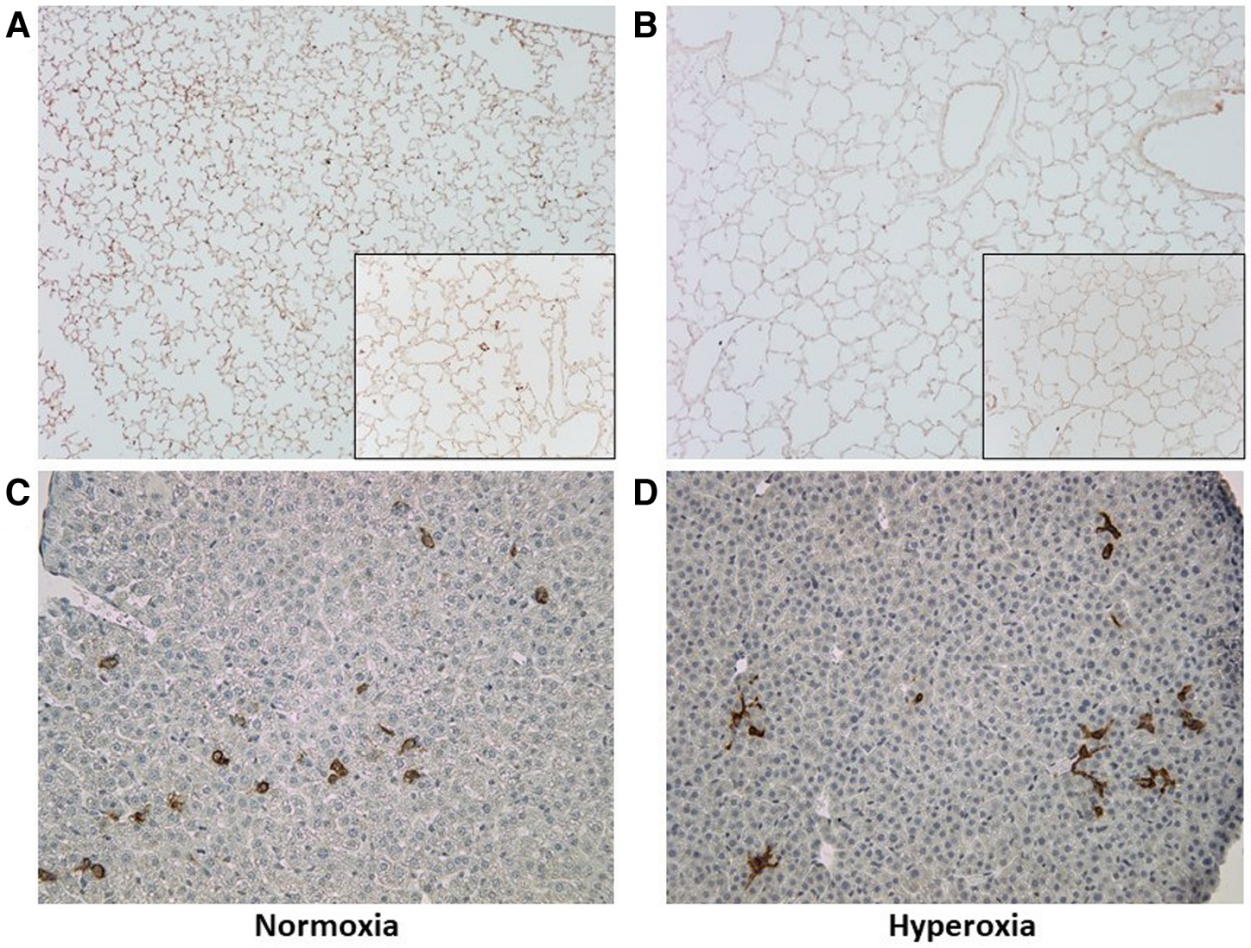
Representative lung and liver tissue sections with anti-human CD45 antibody staining. (**A,B**) Lung tissue sections at P14 demonstrating changes in alveolar structure and distribution of human CD45+ stained cells after normoxia (**A**) versus hyperoxia (**B**); images taken at 10× with 20× inlay. (**C,D**) Liver tissue section at P14 after normoxia (**C**) versus hyperoxia (**D**); images taken at 10×.

The MISTRG mouse lungs harvested at P14 were stained for human CD45 to identify human hematopoietic cells that could have migrated to the lungs. Figure 7 shows the patient-specific profiles of human CD45+ cell counts in the MISTRG lungs and liver at P14 after injection with monocytes from the 10 patients at P0. While the median cell counts varied substantially among patients, there was a significantly decreased presence of human lung cells in mice injected with monocytes from very preterm infants (patients 7 and 10) as compared with the 34 week infant with preeclampsia exposure (patient 3). These patient-specific differences were not seen in hyperoxia-exposed mice. In room air-exposed but not hyperoxia-exposed mice, median human CD45+ cell count in the lungs was higher in mice transplanted with monocytes from preeclamptic births (Table 2). Human CD45+ counts in murine livers did not vary among patients, or with hyperoxia exposure, unlike CD45+ cell counts in the lungs which was decreased with hyperoxia (6.0 ± 0.8 vs. 28.0 ± 12.6 cells per lung field for hyperoxia versus normoxia respectively; *P* = 0.005). The differences in lung field counts were likely due to the alveolar simplification of hyperoxia exposure. When calculating the ratio of lung cell count to total CD45+ cells in lung + liver fields combined, the observed differences with hyperoxia and normoxia exposure were highly variable and no longer significant (Figures 7E,F). Figure 8 shows representative lung (Figures 8A,B) and liver (Figures 8C,D) tissues from mice exposed to hyperoxia versus normoxia.

### Human plasma cytokines and chemokines define the microenvironment of the fetal monocytes

The average levels (assayed in duplicate) of all 30 analytes according to the 10 patients are shown in Table 3. Of the 30 analytes measured, 15 were consistently (≥90%) within the limit of detection. These included FGF-2, eotaxin, FLT-3l, Fractalkin, G-CSF, IL-1RA, IL-6, IL-8, IL-12p40, IL-12p70, IP-10, MCP-1, MIP-1β, TGFα, TNFα. In stepwise linear regression models adjusted for log-transformed GA, infant sex and preeclampsia status, 4 analytes had significant associations with multiple lung parameters: MCP-1, G-CSF, IL-8 and MIP-1β (Table 4). Upon further adjustment for the multiple biomarker comparisons, MCP-1 and MIP-1β were positively correlated with CD45+ lung cell counts while IL-8 was negatively correlated. Upon stratification by hyperoxia exposure (yes/no) these associations were modified: In models restricted to the normoxia group, MCP-1 was positively associated with alveolar count (*P* < 0.001), and negatively associated with perimeter and area (*P* < 0.01).

**Table 3:** Cord blood plasma levels (pg/mL) of the 30 analytes measured by multiplex immunoassay.

Patient #	1	2	3	4	5	6	7	8	9	10
EGF	<3.2	22.48	21.52	<3.2	4.50	<3.2	24.10	27.56	9.73	42.59
Eotaxin	21.22	24.55	75.81	30.61	23.39	19.27	58.30	24.74	19.91	29.63
FGF-2	10.16	37.47	18.32	23.01	ND	129.11	1,201.75	251.33	73.58	55.46
FLT-3l	7.45	17.41	22.85	16.38	1.31	12.26	32.56	15.57	19.67	37.25
Fractalkine	121.24	55.20	116.94	133.02	58.17	83.89	120.86	140.14	66.45	138.27
G-CSF	87.38	<4.8	197.37	106.37	79.07	156.49	2,808.40	323.40	18,544.69	318.40
GM-CSF	ND	<2.6	<2.6	ND	<2.6	<2.6	ND	<2.6	<2.6	<2.6
IFN-γ	<1.3	<1.3	<1.3	<1.3	<1.3	<1.3	<1.3	<1.3	<1.3	<1.3
IL-1α	3.80	1.57	6.95	24.84	ND	ND	40.48	1.99	14.86	12.30
IL-1β	0.17	0.90	23.58	ND	<1.6	<1.6	7.84	0.17	2.17	3.60
IL-1RA	71.11	126.79	714.35	1,562.66	93.20	15.02	10,441.25	330.65	3,541.34	1,531.55
IL-2	0.11	<0.6	0.12	0.57	<0.6	<0.6	0.61	<0.6	<0.6	0.10
IL-3	0.60	<1.3	0.24	2.12	<1.3	<1.3	0.90	0.39	<1.3	0.28
IL-4	<0.6	<0.6	0.73	1.00	0.50	<0.6	0.56	<0.6	<0.6	0.89
IL-6	14.89	0.83	79.66	35.87	7.38	14.91	191.89	14.62	3,923.95	51.90
IL-8	0.93	0.45	43.13	14.81	2.23	7.64	275.69	43.47	59.90	69.21
IL-10	11.89	<2.6	17.58	12.62	26.96	6.21	19.39	9.47	5.82	<2.6
IL-12 (p40)	487.67	132.98	108.00	295.65	75.17	304.92	191.25	319.51	305.41	92.86
IL-12 (p70)	1.39	1.67	4.45	2.02	1.06	1.05	2.39	2.38	1.56	2.63
IL-13	<6.4	ND	19.41	ND	<6.4	<6.4	6.16	14.58	3.84	20.51
IL-17A	<1.3	<1.3	1.01	<1.3	<1.3	<1.3	<1.3	0.25	<1.3	ND
IP-10	<2.6	39.38	388.16	13.25	49.17	18.40	50.92	497.92	151.11	88.22
MCP-1	148.17	93.41	1,310.10	924.83	158.60	356.98	815.10	224.56	235.13	290.21
MCP-3	10.81	ND	25.85	14.96	<8.0	<8.0	18.70	15.08	4.47	27.26
MIP-1α	<3.2	<3.2	21.71	20.71	<3.2	<3.2	35.15	18.44	12.97	35.68
MIP-1β	12.75	44.68	66.47	43.57	12.23	23.05	38.27	29.16	59.45	123.10
TGF-α	2.26	2.31	2.19	7.97	2.26	2.57	2.99	2.17	2.33	2.89
TNF-α	19.36	20.40	29.96	24.61	13.90	17.46	29.25	45.72	76.48	33.93
TNF-β	<1.6	<1.6	1.14	<1.6	<1.6	<1.6	ND	0.94	10.73	2.54
VEGF-α	<2.6	<2.6	ND	<2.6	14.54	<2.6	48.21	59.32	171.37	338.13

Cord blood plasma levels measured by Luminex multiplex immunoassay. Values reported as picogram/ml and are the average of 2 assays performed in duplicate.

EGF, epidermal growth factor; FGF, fibroblast growth factor; FLT, Fms-related tyrosine kinase; G-CSF, granulocyte colony stimulating factor; GM-CSF, granulocyte-macrophage colony-stimulating factor; IFN, interferon; IL, interleukin; IP, interferon gamma-induced protein; MCP, monocyte chemoattractant protein; MIP, macrophage inflammatory protein; TGF, transforming growth factor; TNF, tumor necrosis factor; VEGF, vascular endothelial growth factor.

ND = not available (level was not detected by the assay); levels “<” indicate the calculated value was below the limit of detection for the assay (see MILLIPLEX® Human Cytokine/Chemokine/Growth Factor Panel HCYTA-60K for reference values and ranges).

**Table 4:** Correlations in beta-coefficient (95% CI) between human cord blood plasma cytokine/chemokine levels and humanized mouse lung parameters.

	MCP-1 β-coef (95% CI)	G-CSF β-coef (95% CI)	IL-8 β-coef (95% CI)	MIP-1β β-coef (95% CI)
All (*N* = 56)				
Alveolar count	0.13 (−0.25, 0.50)	0.09 (−0.13, 0.30)	−0.28 (−0.67, 0.11)	0.56 (−0.11, 1.22)
Alveolar perimeter	−0.03 (−0.13, 0.07)	−0.01 (−0.07, 0.05)	0.03 (−0.08, 0.13)	−0.13 (−0.31, 0.05)
Alveolar area	−0.13 (−0.38, 0.12)	0.02 (−0.13, 0.17)	0.05 (−0.21, 0.31)	−0.22 (−0.67, 0.23)
Microvessel density	0.23 (−0.08, 0.54)	0.06 (−0.12, 0.24)	−0.23 (−0.56, 0.09)	0.23 (−0.32, 0.79)
Lung CD45+ cell count	1.00 (0.55, 1.45)***	0.03 (−0.23, 0.30)	−1.07 (−1.56, −0.58)***	1.08 (0.31, 1.85)**
Normoxia (*n* = 26)				
Alveolar count	0.29 (0.13, 0.45)***	0.03 (−0.09, 0.16)	−0.15 (−0.35, 0.06)	0.02 (−0.29, 0.33)
Alveolar perimeter	−0.13 (−0.23, −0.04)**	−0.05 (−0.12, 0.02)	0.12 (0.00, 0.24)*	−0.16 (−0.34, 0.02)
Alveolar area	−0.29 (−0.50, −0.09)**	−0.004 (−0.15, 0.16)	0.10 (−0.17, 0.36)	0.04 (−0.37, 0.45)
Microvessel density	−0.13 (−0.27, 0.02)	−0.07 (−0.18, 0.04)	0.18 (−0.01, 0.36)*	−0.09 (−0.38, 0.20)
Lung CD45+ cell count	0.78 (0.04, 1.52)*	0.05 (−0.50, 0.59)	−1.20 (−2.21, −0.19)*	1.57 (0.19, 2.97)*
Hyperoxia (*N* = 30)				
Alveolar count	−0.24 (−0.52, 0.04)	0.01 (−0.13, 0.15)	0.02 (−0.25, 0.29)	0.06 (−0.43, 0.55)
Alveolar perimeter	0.08 (−0.02, 0.17)	0.02 (−0.03, 0.07)	−0.08 (−0.18, −0.01)*	0.01 (−0.15, 0.18)
Alveolar area	0.11 (−0.06, 0.28)	0.09 (0.00, 0.17)*	−0.19 (−0.35, −0.03)*	0.09 (−0.20, 0.38)
Microvessel density	0.34 (0.03, 0.66)*	0.02 (−0.14, 0.18)	−0.22 (−0.52, 0.08)	−0.16 (−0.70, 0.37)
Lung CD45+ cell count	0.96 (0.43, 1.50)***	−0.05 (−0.33, 0.23)	−0.71 (−1.24, −0.18)*	0.16 (−0.74, 1.06)

MCP-1, monocyte chemoattractant protein-1; G-CSF, granulocyte colony stimulating factor; IL-8, interleukin-8; MIP, macrophage inflammatory protein 1-β (CCL4).

All parameters log transformed to approximate normal distribution. Models adjusted for gestational age in weeks (log transformed), infant sex, and preeclampsia status, and for the multiple biomarker comparisons.

* *P* < 0.05.

** *P* < 0.01.

*** *P* ≤ 0.001.

### Human CD206 alveolar macrophages are absent in MISTRG lungs at P14, but emerging at P28 after recovery in normoxia

**Figure 9:**
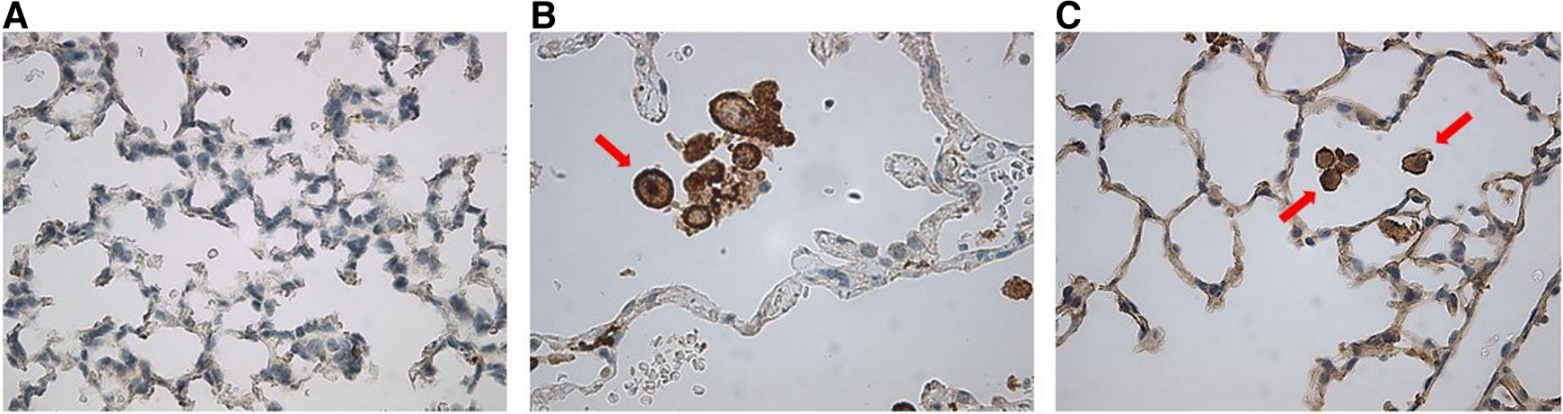
Anti-human CD206 stained MISTRG mouse lung slides showing (**A**) absence of alveolar macrophages at p14 as compared with (**B**) positive human control. (**C**) At p28, there was emergence of positively-stained cells with prominent nuclei similar to human controls, as indicated by red arrows.

**Figure 10:**
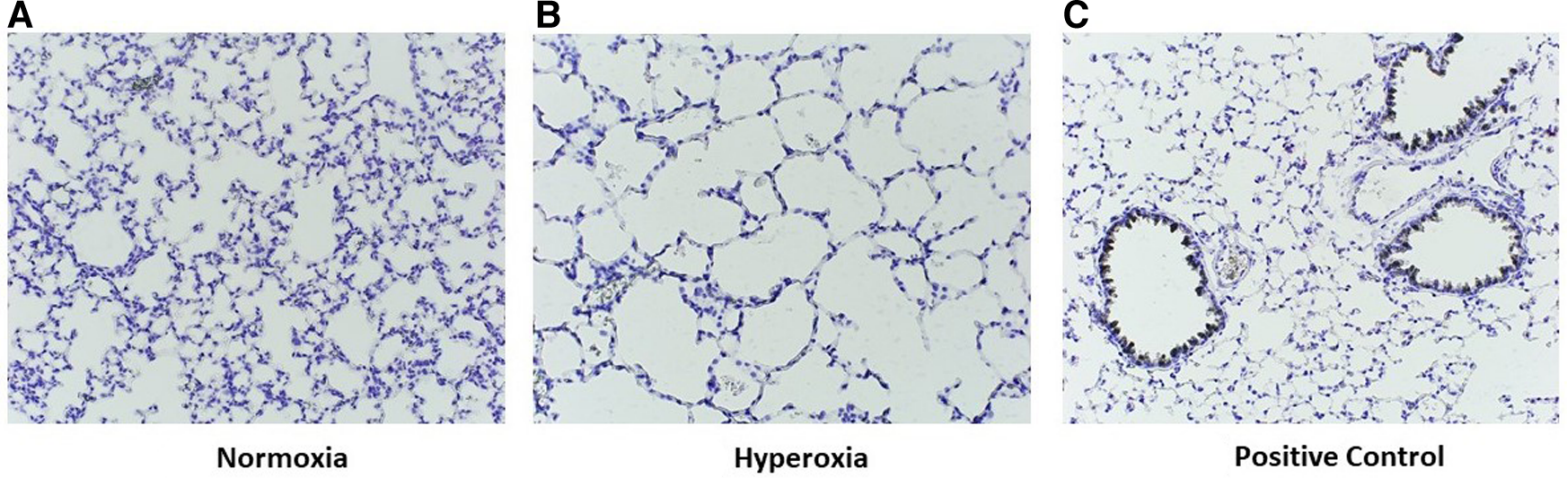
Representative images of p14 lungs with F4/80 immunostaining. Lungs harvested at p14 were stained with F4/80 to identify murine lung macrophages. Note the absence of positive staining in both normoxia-exposed (**A**) and hyperoxia-exposed (**B**) lungs as compared with positive control lung from an adult wildtype non-humanized mouse lung tissue (C57BL/6J, compliments of Dr. Eniko Sajti) (**C**). For all slides, biotin labelled secondary antibody used, with counterstain incubation time 30 s Antibody dilution for both primary and secondary was 1:200. Streptavidin-HRP dilution 1:1500. Images taken at 20×.

Staining for anti-human CD206 antibody did not yield positively stained cells, as shown in representative samples of lungs at p14 (Figure 9A) as compared with positive human control samples (Figure 9B). Therefore, we studied a subsequent group of mice similarly transplanted with human monocytes (p0) and exposed to hyperoxia (p0–p14) and allowed to recover in room air (p15–p28). Lungs harvested at p28 revealed positively stained cells in several alveoli with morphology consistent with alveolar macrophages (Figure 9C). Additional studies of IHC staining for anti-mouse F4/80 in 10 representative mouse slides from each of the 10 patient monocyte cell lines confirmed absence of mouse macrophages in lungs at P14 (Figure 10).

**Figure 11:**
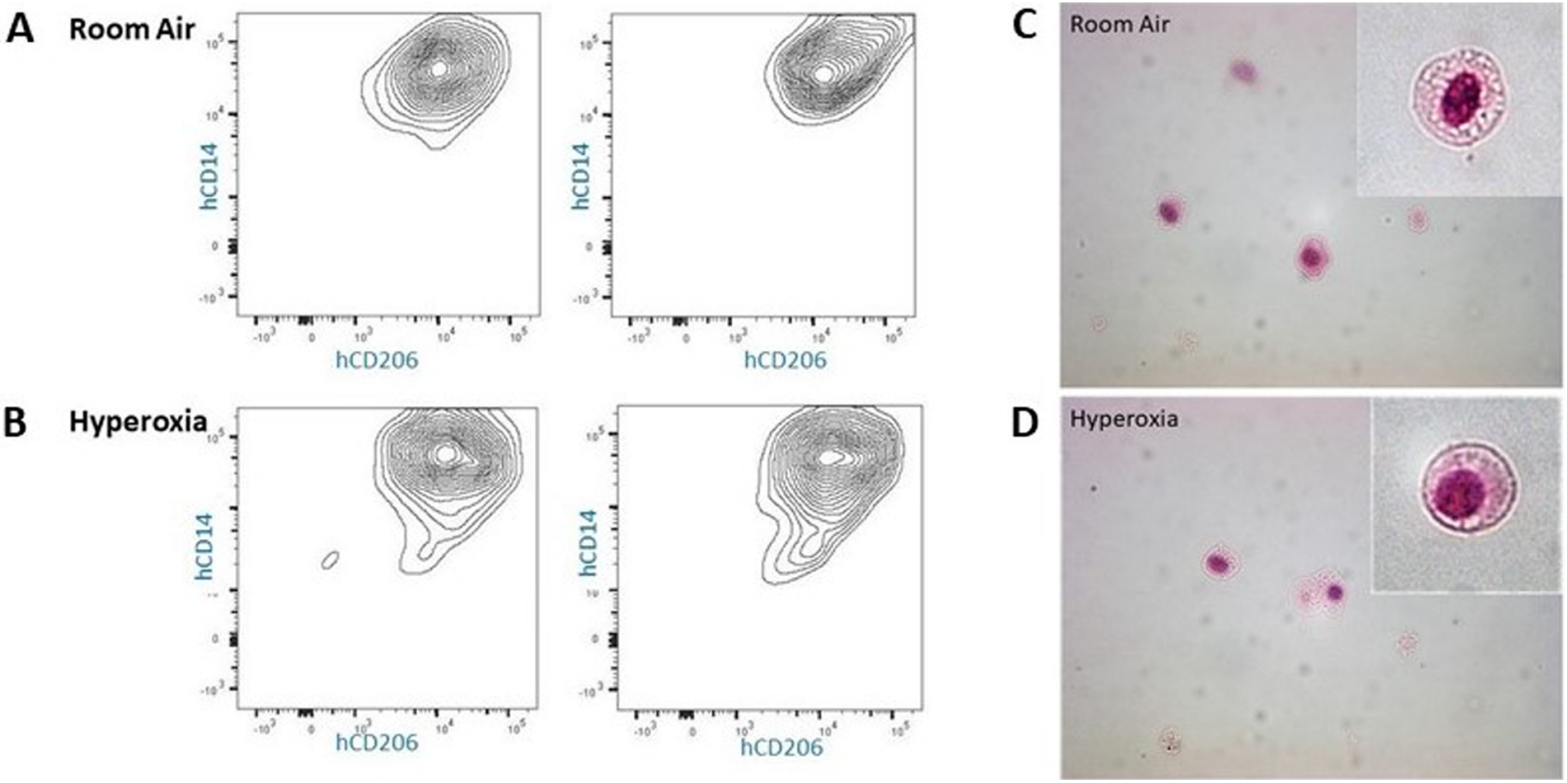
Cytospin images (40×) of hCD14^+^/hCD206^+^ lung cells isolated from p56 MISTRG lungs. (Panel **A,B**) Flow cytometry gating by HLA-DR^+^/mouseCD45^−^ followed by FACS for isolation of human CD14^+^/human CD206^+^ cells from lungs exposed to room air or hyperoxia ×14 days followed by recovery in room air until 8 weeks of age. (Panel **C,D**) Cell surface markers and morphology (inlay) confirm the presence of human alveolar macrophages at p56 in MISTRG mice exposed to room air and early hyperoxia.

To further explore the morphology and presence of human lung cells at p56, a subset of mice were allowed to recover in room air an additional 28 days, after which lung tissues were harvested at p56 and underwent flow cytometry/FACS sorting to isolate HLA-DR+, hCD206+, hCD14+, mCD45- viable cells which were stained for H&E following cytospin. This process confirmed the presence of human cells with morphology of alveolar macrophages, both in room air and hyperoxia-exposed mice at p56 (Figure 11).

### RT-PCR studies of p14 lungs support paucity of human cells in the lungs expressing MCP-1, VEGF-A and MIP-1β

**Figure 12:**
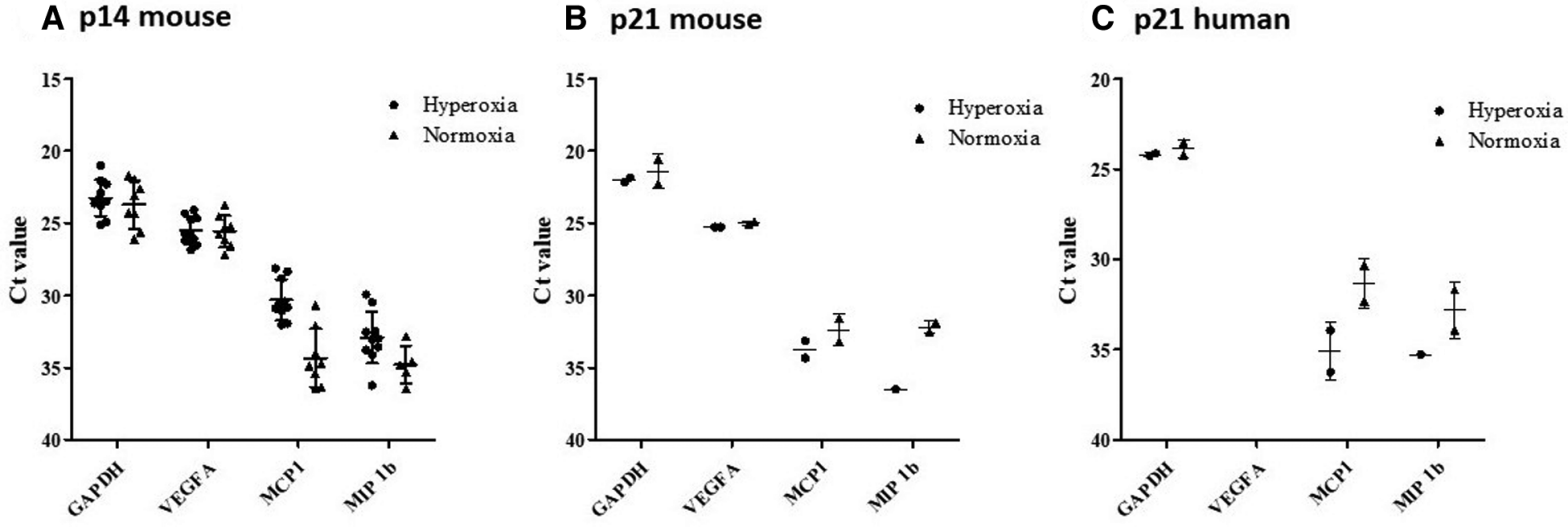
RT-qPCR results from MISTRG lungs at p14 showing gene expression changes with hyperoxia. According to plotted Ct values (*y*-axis) VEGF-A for normoxia and hyperoxia were not remarkably different. Whereas, MCP-1 and MIP-1B lower Ct values were observed in hyperoxia as compared to normoxia, indicating relatively higher levels of RNA (Panel **A**). Relative to p14, there was down-regulation (as noted by higher Ct value) of mouse MCP-1 and MIP-1B but not mouse VEGF-A after recovery in normoxia at p21 (Panel **B**). There was emergence of human MCP-1 and MIP-1B expression at p21, which was not seen at p14 (Panel **C**).

RT-qPCR results indicated significant levels of gene expression of mouse MCP-1, VEGF-A, and MIP-1β in all 18 representative lung samples harvested at p14 (9 normoxia-exposed and 9 hyperoxia-exposed). There was minimal detection of human gene expression at p14 (data not shown), but upregulation of mouse gene expression for all 3 genes with hyperoxia exposure (Figure 12A). In addition, 4 representative lung samples harvested at p21 in additional mouse experiments were analyzed. Relative to p14, there was down-regulation (as noted by higher Ct value) of mouse MCP-1 and MIP-1β but not mouse VEGF-A after recovery in normoxia at p21 (Figure 12B). There was emergence of human MCP-1 and MIP-1β at p21 (Figure 12C).

## Discussion

In this study of a humanized mouse model of neonatal hyperoxia-induced lung injury, we found that newborn adoptive transfer of cord blood-derived fetal monocytes resulted in modest changes in alveolarization at p14. Unexpectedly, we found early signs of improved pulmonary microvessel density with human monocytes versus placebo injection at p0, suggesting a novel role of human monocytes in early pulmonary vascularization. Distinct patterns were also discovered in pooled patient analyses when GA and preeclampsia status of patient-specific monocytes were taken into account. While some of our findings support previous reports of human monocyte adoptive transfer in murine models (8), our study utilized a novel model genetically designed to support human monocyte transplantation, survival and potential for engraftment, through knock-in of human GM-CSF and other human cytokines (6). Leveraging the unique features of this humanized mouse model, we also took into account key human features of pregnancy that are associated with known perinatal risk factors for BPD. We found that monocytes derived from births complicated by preeclampsia resulted in features consistent with accelerated alveolarization (increased alveolar count, decreased area). While the effects of hyperoxia were largely independent of the pregnancy features, many of the findings in alveolarization and vascularization were in mice exposed to room air. This provides the first evidence that there are inherent features of human monocytes that may play a role in early lung development. These findings have important implications for our understanding of BPD pathogenesis.

The role of alveolar macrophages in early lung development and chronic lung diseases such as BPD has been increasingly studied, with growing recognition that monocyte precursors play a pivotal role in lung injury and repair (21–23). Studies have shown a beneficial effect of human cord blood monocyte administration postnatally (p7) in a double-hit model of BPD (prenatal hypoxia followed by postnatal hyperoxia) (24), but these and other animal models of BPD have not been genetically modified to minimize human cell rejection. Mills and colleagues treated severe combined immunodeficient (SCID) mice with monocytes from cryopreserved cord blood via intravenous route and found mild improvement in alveolarization, lung compliance/elastance and decreased methacholine-induced bronchial hyperreactivity at 8 weeks (8). In contrast, we focused our investigations on a much earlier stage of alveolarization (p14) and used a mouse model (MISTRG) that specifically supports human monocyte-macrophage survival and engraftment. We administered monocytes via intrahepatic injection to more closely recapitulate the source of fetal monocytes *in vivo* and found not only robust survival of the mouse host but of circulating human monocytes at p14. Unlike typical animal models of BPD, we also describe detailed clinical characteristics of the patients from which the monocytes were derived and took these factors into consideration in our analysis. Measurement of cytokines and chemokines in human cord blood plasma from these births was also performed to understand the human immune cell microenvironment at the time of monocyte collection (i.e., birth).

We had hypothesized to find mostly changes in alveolarization with monocyte versus placebo treatment. Rather, more prominent and intriguing differences were seen in the vascular parameters. Despite only modest differences in alveolar morphology that were independent of hyperoxia exposure (Figure 2), mice treated with human monocytes had higher microvessel density than PBS-treated mice in room air (Figure 3A). This suggests that fetal monocytes may play a role in supporting early vascularization at baseline, an effect that was modified by hyperoxia such that median microvessel density in monocyte-treated mice fell to levels similar to PBS-treated mice after exposure to 85% oxygen × 14 days (Figures 3A, 4).

Another unexpected finding was the baseline levels and changes in Fulton's index, a well-characterized measure of pulmonary hypertension in rodent models (25). In PBS-treated mice exposed to hyperoxia × 14 days, for example, mean Fulton's indices typically rise from 0.20 ± 0.01 to 0.35 ± 0.04 (25), indicating an elevated RV/LV + S ratio that usually accompanies a decrease in microvessel density. While the indices in our study were quite variable with some outliers >0.4, there was an overall decrease in Fulton's index with hyperoxia as compared with baseline. The decrease was statistically significant in the monocyte-treated group, supporting the hypothesis that fetal monocytes may incur a protective effect against pulmonary hypertension. We speculate that this effect may be independent of early pulmonary vascular development as the microvessel density was decreased in this group with hyperoxia. Of note, the base line Fulton's indices in the MISTRG mice were somewhat higher at baseline than other, non-humanized mouse models (19, 26), suggesting that the absence of early lung macrophages in p14 MISTRG mice (Figure 10) may play a role in development of pulmonary hypertension. There is recent emerging evidence that alveolar macrophages may contribute to pulmonary hypertension (27). To our knowledge, the vascular findings reported here in the MISTRG model of neonatal hyperoxia-induced lung injury have not been previously reported. Thus, further investigation leveraging this model may elucidate mechanisms of distinct vascular endotypes of BPD, such as BPD-associated pulmonary hypertension (28).

MISTRG mice were first described in 2014, developed by Rongvaux and colleagues to model the human immune system in a wide range of organ systems and disease states (6). The unique features of this model include knock-in of human macrophage colony-stimulating factor (M-CSF) and GM-CSF—two cytokines essential for macrophage development (29, 30), as well as expression of human cytokines interleukin-3 (IL-3) and thrombopoietin (TPO) in a immunodeficient Rag2^−/−^Il2rg^−/−^ background, and signal regulatory protein alpha (SIRPα) to establish mouse-to-human phagocytic tolerance (6). This supports a highly permissive microenvironment for human hematopoiesis in a mouse host (3). A growing number of studies have used MISTRG mice to study various disease states (31–35). To our knowledge none have been reported to date on the effects of neonatal hyperoxia, prematurity or preeclampsia. Evren and colleagues are the first to provide a comprehensive report of the early course and fate of human CD34^+^ hematopoietic stem and progenitor cells (HSPCs) in MISTRG lungs (3). In a series of adoptive transfer and fate mapping studies, they showed that HSPCs migrate to lung tissue and give rise to human interstitial and alveolar macrophages. They reported that HSPCs give rise to all types of human lung monocytes and macrophages, and that classical CD14^+^CD16^−^ monocytes (the most abundant subpopulation in our cord blood samples) appear >3 weeks postnatally in the mice. In fate mapping studies, classical monocytes were shown to be the precursors of human lung macrophages—with a predominance of alveolar macrophages. However, similar to our findings upon anti-human CD206 staining, they found a paucity of CD206+ cells at <3 weeks postnatally, and an emergence of these as human alveolar macrophages at 8 weeks suggesting that CD14^+^ monocytes are at most the precursors to adult alveolar macrophages.

The CD45^+^ cells present in lung tissue at p14 in our studies most likely represent circulating hematopoietic human cells that have migrated to the lungs, however the overall paucity of these cells in the lung and their location in vascular rather than alveolar regions suggests that the original CD14^+^ fetal monocytes derived from cord blood and transplanted at P0 are not immediate early precursors to alveolar macrophages in the perinatal period. These findings are consistent with a more recent study by Evren, et al, in which they identified a distinct lineage of circulating CD116^+^CD64^−^ fetal macrophage precursors that originate in the fetal liver and readily migrate to the perinatal lung in the MISTRG model, populating the lungs at <7 weeks postnatally as functional alveolar macrophages (36). In conjunction with these recent findings, our data alternatively suggest a pulmonary vascular and/or paracrine effect of CD14^+^ monocytes on lung alveolarization, in which CD14^+^ monocytes remain in circulation and express differential gene expression according to their exposure to acute inflammation or vascular dysfunction mediated by the placenta (7).

Our data showed that hyperoxia resulted in significantly reduced median CD45+ cell counts in the lungs, but not the liver (Figures 7, 8). Overall, these data and to a lesser extent the lung morphometry data were quite variable with small numbers of animals in certain groups given the limitations in timing matings, litter size and availability of cells for injection which may have been due to inherent properties of cord blood monocytes in human infants and their birth characteristics (e.g., fewer monocytes were recoverable from lower gestational age and/or preeclamptic births). Thus, pooled analysis of the patients according to these birth characteristics was necessary. As such, both individual and pooled analysis suggests that preeclampsia could be a driver of increased median cell count in the lung, and that this effect may be modified by hyperoxia (Figure 7 and Table 2). Mechanisms linking preeclampsia to accelerated alveolarization have not been completely described, but recent data suggests that accelerated placental aging, a feature of preeclampsia (37, 38), is associated with the certain vascular phenotypes of BPD (39), and that reversal of accelerated lung aging characterized by cell senescence and apoptosis could attenuate hyperoxia-induced lung injury (40). Britt and colleagues reported reduced levels of monocyte chemoattractant protein (MCP-1) in bronchoalveolar lavage fluid at p14 in hyperoxia-exposed mice, suggesting a possible mechanism by which hyperoxia is associated with decreased human CD45+ cells in lungs at p14 in our MISTRG model (41). Our findings also suggest that the hypoxic insult of preeclampsia alone may be a significant driver of *increased* migration of monocytes to the lung. These findings are consistent with previous *in vitro* observations of hypoxia-induced directed migration of human monocytes (42). While the effects of subsequent hyperoxia following hypoxia on monocyte migration are not well-understood, double-hit models of monocyte replenishment at p7 in mice support our findings that hypoxia-hyperoxia leads to decreases in circulating lung monocytes (24).

As gestational immaturity is an independent risk factor for BPD, it is not surprising that we saw differences in monocyte function among the 3 gestational age subgroups. Delayed, rather than accelerated alveolarization appeared to be associated with adoptive transfer of monocytes from very preterm births. In our humanized mouse model, the pattern of alveolar simplification with lower gestational age was modified by hyperoxia such that the differences between the gestational age subgroups were lost (Table 2). However, the decrease in CD45+ cell count with hyperoxia was preserved (Figure 7), suggesting that monocytes engraft differently with hyperoxia when certain pathologies (extreme prematurity, intrauterine inflammation or placental vascular dysfunction) are also present. It cannot be determined from our study whether the decrease in CD45+ cells contribute to delayed alveolarization, and further studies of the function of these circulating lung cells are needed. In a separate study of cord blood monocytes derived from this same patient cohort, bulk RNAseq revealed distinct gene expression profiles mediated by inflammatory versus vascular processes (7). These findings, coupled with single cell RNAseq studies conducted by Evren et al, that identified distinct developmental pathways from circulating MNCs to lung macrophages (3) support a pivotal role of circulating fetal-derived monocytes in early lung development and subsequent BPD.

Certain findings of cord blood cellular count and plasma contribute to our understanding of monocyte interactions and their microenvironment. It is important to note that across all types of patient samples in our cohort, classical monocytes were the most predominant at birth, and that differences in proportion of monocyte subsets may play a role. Consistent with Evren et al, it is likely that the classical subpopulation represents the predominant source of human CD45+ cells present in circulation at p14, which are possible precursors to the CD206+ alveolar macrophages present after 3 weeks (Figure 9). Our humanized mice findings coupled with recent data from the larger cohort support the emerging role of atypical, non-classical monocytes in vascular processes by which preeclampsia is associated with distinct endotypes of BPD (43, 44).

In multiplex analysis of corresponding cord blood plasma samples obtained at the time of monocyte isolation at birth, we found that MCP-1, MIP-1β and IL-8–chemokines known to be induced by monocytes in various disease states and are key regulators of migration and infiltration of monocytes and macrophages (45–47)—were differentially associated with several parameters of alveolarization, microvessel density, and circulating human CD45+ cells in the lungs. These chemokines, in addition to G-CSF, are known to play an interactive role in neutrophil recruitment and function (48, 49). Yet it remains unclear whether and which proteins present in plasma represent expression from circulating monocytes and whether human monocyte expression is similar after transplantation into MISTRG mice. In the RT-qPCR studies (Figure 12) human gene expression, but not mouse gene expression, correlates with presence of human cells in the lungs. These findings suggest, albeit preliminarily, that monocytes after birth continue to contribute to their microenvironment and may have paracrine effects on the lungs (perhaps from the liver) in the absence of human lung macrophages.

There were several limitations in this study. Firstly, a much larger sample of patients is needed to more completely understand the diversity of monocytes and the influence of perinatal risk factors. At the onset of this study, we had originally hypothesized that monocytes would readily engraft in the MISTRG mice and influence lung development via monocyte-derived alveolar macrophages. Emerging literature has since shown that these alveolar macrophages do not appear until much later in development, beyond the perinatal period, which is supported by our findings. But the finding that monocytes may accelerate early microvessel development before the appearance of macrophages generates new hypothesized mechanisms of perinatal lung vascular development influenced by innate immune mechanisms. Further investigations of how vascular changes arise in the lungs despite the lack of transplanted immune cells in early development are needed, as these studies may redirect our focus towards other cell types or perhaps paracrine effects on the lungs from distant or circulating monocytes. As the MISTRG model has been used in various studies of adult lung disease, there is a steep learning curve for leveraging the unique aspects of this model for more mechanistic interrogation of BPD—a developmental lung disease that is multifactorial and highly complex.

As our cord blood monocyte archive expands, we hope to interrogate more samples from patients who develop BPD. A significant challenge in using human infant-specific cord blood monocytes is that the process requires cryopreservation of the monocytes, sometimes for longer periods than traditionally done, to allow well-matched patient samples to be adoptively transferred in sequential studies with well-coordinated timed matings. Variations in litter size and patient-specific factors led to variations in the number of pups injected for each exposure group. There are also limitations in the volume of blood and cells that can be collected from extremely preterm infants, requiring pooled analysis of patients according to their birth characteristics. While our preliminary studies showed excellent cell viability and function in gene expression studies using the methods described in this study, the workflows to optimize viability and improve cell recovery are critically important for the success of each experiment, and to study more litters of MISTRG mice simultaneously to minimize batch effects. Lastly, another important limitation of this study is that we have yet to study the long-term effects of monocyte injection in larger samples, in particular the sustained effects of early microvessel development after recovery in normoxia. If sufficient human lung macrophage populations are successfully established using the above processes, limitations of alveolar proteinosis in adult mice may be overcome so that these further timepoints can be investigated. Methods for following the course and trafficking of immune cells can be employed in real-time using recently developed immunophenotyping technologies. In addition, studying other organ systems and larger scale analysis of the lungs, such as RNAseq and proteomics, will be highly informative.

In summary, we conducted this study to test the hypothesis that fetal monocytes influence early lung development in a novel humanized mouse model of hyperoxia-induced lung injury. We found that fetal monocytes directly influence pulmonary vascular development, despite the absence of alveolar macrophages in the neonatal period of MISTRG mice. Furthermore, the vascular effects induced by monocytes are inhibited by hyperoxia, but may be independent of pulmonary hypertension. The above study describes a novel *in vivo* experimental model of BPD by which human monocytes and their associated clinical and perinatal characteristics can be investigated for effects of neonatal hyperoxia on early lung development. Elucidation of the mechanisms by which fetal monocytes, programmed by *in utero* processes such as vascular dysfunction, influence lung injury and repair is essential for developing more targeted, patient-specific approaches in the management of multifactorial BPD.

## Data Availability

The raw data supporting the conclusions of this article will be made available by the authors, without undue reservation.
